# Research Progress on the Application of Anthocyanins in Food Preservation

**DOI:** 10.3390/foods15214009

**Published:** 2026-11-09

**Authors:** Shujuan Zheng, Yu Lu, Xinyu Wang

**Affiliations:** College of Biological and Food Engineering, Anhui Polytechnic University, Wuhu 241000, China; 13856433216@163.com (Y.L.); 13376218598@163.com (X.W.)

**Keywords:** anthocyanins, antioxidant activity, antibacterial activity, food preservation

## Abstract

Anthocyanins are a class of naturally water-soluble pigments widely present in plants and are highly promising natural preservatives. Anthocyanins exert excellent antioxidant and antibacterial activities. Anthocyanins effectively inhibit lipid peroxidation and protein oxidation, suppress microbial reproduction, and delay food spoilage. The excellent pH response characteristic of anthocyanins enables real-time indication of food freshness. This review reveals the sources and antioxidant and antibacterial properties of anthocyanins; research progress on the use of anthocyanins in preserving fruits and vegetables, meat products, aquatic products, and processed foods; and looks forward to the development direction of anthocyanins in the field of food preservation to promote the practical application of anthocyanins in food preservation packaging materials.

## 1. Introduction

Foods are susceptible to losses during storage due to limitations in packaging methods, transportation conditions, and harvesting methods [1]. Lipid oxidation, protein oxidation, and microbial growth and reproduction are prone to occur during food preservation, causing food spoilage [2,3]. Food freshness is sometimes difficult to assess by observation.

Anthocyanins are a class of water-soluble flavonoids widely present in plants, endowing them with rich colors. Anthocyanins occur widely in blueberries, grapes, aronia, pomegranates, elderberries, lingonberries, raspberries, and other plants [4,5,6,7]. Anthocyanins can be degraded [8]. The safety of anthocyanins depends on the concentration used, the exposure dose, the specific compounds or extracts, other substances added during practical application, and the application process. Anthocyanins have antioxidant and antibacterial properties that effectively inhibit food spoilage. Moreover, the pH-responsive, color-changing properties of anthocyanins enable them to indicate food freshness in real time. Hence, anthocyanins are promising materials for preservative packaging.

This review covers the sources, extraction methods, and antioxidant and antibacterial mechanisms of anthocyanins, and focuses on their role as active ingredients in intelligent active packaging for food preservation and freshness indication, providing new strategies for applying anthocyanins in food preservation packaging.

## 2. Literature Search and Study Selection Methodology

**Figure 1:**
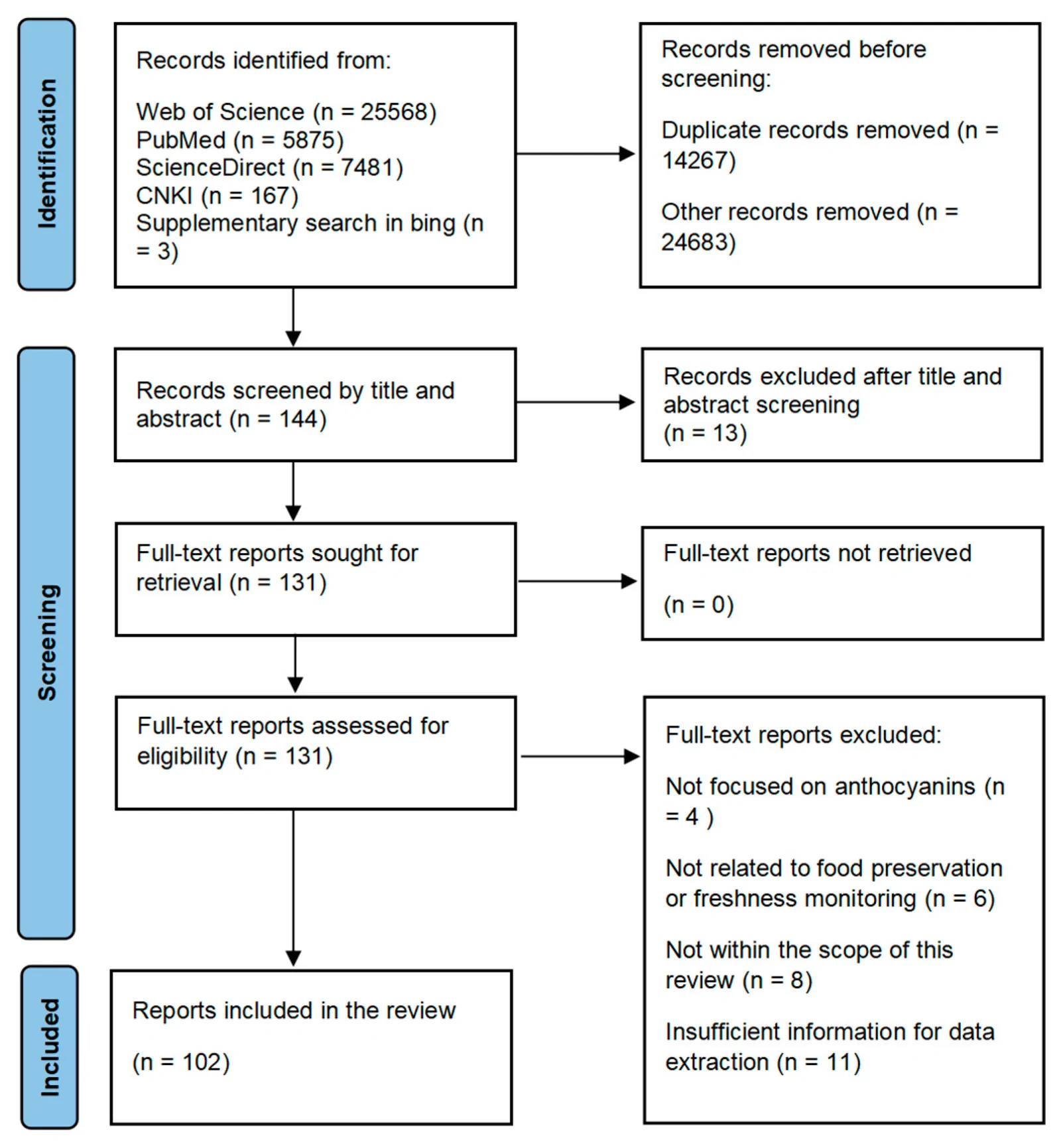
Adapted PRISMA-style flow diagram illustrating the identification and selection of studies for this comprehensive review.

A comprehensive review was conducted to recent advances of anthocyanins in food preservation. This study searched the ScienceDirect, Web of Science, and PubMed databases using keywords and title and abstract searches, and used the Bing search engine for supplementary searches. Based on the articles’ content, we conducted a theme-based keyword search. First, a search was conducted on the basic properties and functional activities of anthocyanins, and the literature search period was set from January 2017 to March 2026. In addition, early classic studies with foundational significance were included through reference tracking. “Anthocyanin” was used as the core keyword and combined with “source”, “structure”, “extraction”, “antioxidant activity”, and “antimicrobial activity” to retrieve studies on these topics. For research on anthocyanins in food preservation, we included representative studies from the past six years to ensure timeliness and novelty. The core search combination was “anthocyanin” and “food preservation”, and related keywords were further expanded according to food types, including fruit, vegetables, meat (chicken, beef, mutton, and pork), aquatic products, and processed foods. To retrieve more studies on processed food systems, we also conducted a supplementary search in the CNKI database. Based on this article’s research topic, we screened the retrieved literature. We included studies on anthocyanin sources, structure, extraction, antioxidant and antimicrobial activity, and food preservation applications. These included original research papers published in peer-reviewed journals and representative review articles. Duplicate publications, conference abstracts, and studies involving research subjects outside the scope of this review were excluded. For food applications, studies that did not evaluate preservation efficacy or freshness-indicating performance, or did not clearly identify the functional contribution of anthocyanins were also excluded. We organized the retrieved documents using reference management software and screened them based on their titles, abstracts, and full texts. A total of 102 documents that met the research objectives were finally included (Figure 1).

## 3. Sources of Anthocyanins

**Figure 2:**
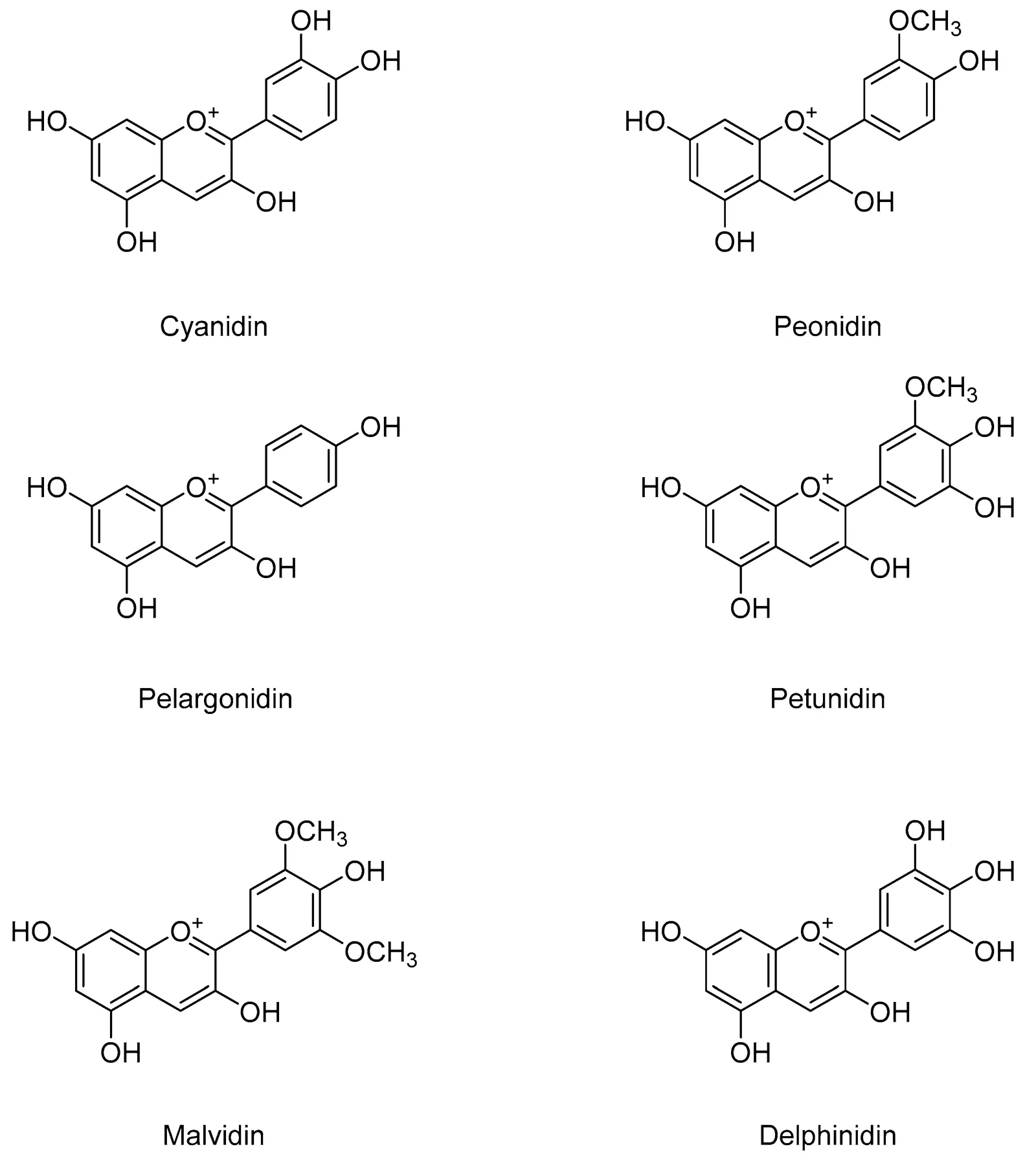
Structural components of the main anthocyanidins [13].

Anthocyanins are a class of food coloring compounds belonging to the flavonoid class, widely present in flowers, vegetables, fruits, leaves, and grains [9]. It has been found that there are at least 650 anthocyanins [10]. Anthocyanins are a class of water-soluble flavonoids formed by the combination of anthocyanidins with one or more sugar molecules through glycosidic bonds. Glycosylation usually occurs at the C3 position but may also occur at the C5 or C7 position. Their basic structural backbone is the 2-phenylbenzopyrylium (flavylium) cation [11]. Common types of anthocyanins include the 3-O-glucosides and the 3,5-O-diglucosides of anthocyanidins [12]. The structures of the six main anthocyanidins are shown in Figure 2 [13]. Anthocyanins are unstable and easily degraded by factors such as pH, light, temperature, and enzyme catalysis [14,15,16,17]. Anthocyanins are highly pH-sensitive and undergo reversible structural transformations. Under strongly acidic conditions, anthocyanins mainly exist as stable flavylium cations and appear red; as the pH increases, they transform into forms such as quinone bases, semiketals, and chalcones. Quinone bases appear purple or blue, while semiketals and chalcones are usually colorless or pale yellow, and their stability decreases under neutral or alkaline conditions [18,19,20]. Ultraviolet light can trigger the photodegradation reaction of anthocyanins [21]. The higher the temperature, the faster the degradation of anthocyanins [22]. Some enzymes such as peroxidase and β-glucosidase can trigger anthocyanin degradation [16].

## 4. Anthocyanins Extraction Methods

Common anthocyanin extraction methods include solution extraction, ultrasound-assisted extraction, and microwave-assisted extraction (Table 1). Solvent extraction is a classic extraction technique based on the principle of “like dissolves like”. Water, methanol, and ethanol are commonly used solvents. For example, anthocyanins were extracted from the fruit skin of *Rhodomyrtus tomentosa* (Ait.) Hassk using 60% ethanol containing 0.1% hydrochloric acid, and the highest concentration of the anthocyanin extract was 4.358 ± 0.045 mg/g. The main anthocyanins in the extracts were delphinidin-3-glucoside, cyanidin-3-glucoside, paeoniflorin-3-glucoside, petunidin-3-glucoside, and malvidin-3-glucoside. When the temperature exceeded 65 °C, longer extraction times decreased anthocyanin yield [23]. Long-term extraction of anthocyanins may lead to degradation [24]. Ultrasonic-assisted extraction is a highly efficient technique that uses cavitation, mechanical vibration, and thermal effects to accelerate solvent penetration and disrupt cell structure. Researchers extracted anthocyanins from purple sweet potatoes using an ultrasound-assisted enzymatic hydrolysis method, yielding a total anthocyanin content of 2.27 mg/g [25]. Microwave-assisted extraction is a separation technique that utilizes the thermal and non-thermal effects of microwaves to promote the dissolution of target components by solvents. Anthocyanins were extracted from *Purple-heart Radish* using microwave-assisted deep eutectic solvents, with the extraction content reaching 6.442 ± 0.135 mg/g. When the extraction temperature exceeded 50 °C and the extraction time exceeded 10 min, anthocyanin decomposition occurred [26].

## 5. Bioactivity of Anthocyanins

### 5.1. Antioxidant Capacity of Anthocyanins

Oxidation is a major cause of food spoilage. The phenolic hydroxyl structure of anthocyanins endows them with strong antioxidant activity. The 2,2′-Azino-bis (3-ethylbenzothiazoline-6-sulfonic acid) (ABTS) free radical scavenging rate of *Dioscorea alata* Linn. anthocyanins at a concentration of 4 mg/mL reached 93.24% [29]. Purple rice extract containing anthocyanins showed potent antioxidant properties. The ABTS free radical scavenging rate was 524.26 ± 4.63 mg Vitamin C equivalent antioxidant capacity/g extract, the IC_50_ of lipid peroxidation inhibition was 19.70 ± 0.31 μg/mL, the IC_50_ of the superoxide anion scavenging was 11.20 ± 0.25 μg/mL, and the IC_50_ of the nitric oxide free radical scavenging was 17.12 ± 0.56 μg/mL [30]. Compared with mustard mutants lacking anthocyanins, anthocyanin-rich mutants exhibited lower oxidative stress levels and stronger antioxidant activity [31]. The antioxidant characteristics corresponding to specific anthocyanins need to be determined based on experimental results.

Anthocyanins are glycosylated derivatives of anthocyanidins. Their aglycone structure is based on a flavylium cation skeleton, consisting of two aromatic rings (rings A and B) and an oxygen-containing heterocyclic ring (ring C), and features a C6–C3–C6 conjugated system [32]. Anthocyanin antioxidant activity is closely related to the distribution of phenolic hydroxyl groups on the B ring, the conjugated structure, and glycosylation. The antioxidant activity of anthocyanins relates to the distribution of phenolic hydroxyl groups on the B ring and the conjugated structure, which facilitate hydrogen-atom and electron transfer, thereby promoting free-radical scavenging. C3 glycosylation can alter the reactive sites and spatial environment of a molecule, resulting in structure-dependent differences in antioxidant activity [33].

### 5.2. Antibacterial Activity of Anthocyanins

The antibacterial mechanisms of anthocyanins involve disrupting cell membrane integrity, interfering with energy metabolism, inhibiting bacterial growth, interfering with amino acid, lipid, and carbohydrate metabolism, and inhibiting DNA replication and repair [13]. For example, the integrity of the cell membrane of *Escherichia coli* O157:H7 was disrupted by anthocyanins isolated from *Aronia melanocarpa* at 0.313 mg/mL. Proteomic analyses showed that the pathway for the anthocyanins to enter cells was through mechanosensitive channels. The anthocyanins increased intracellular reactive oxygen species (ROS) levels, suppressed heat shock proteins, and interfered with intracellular lipid, carbohydrate, and amino acid metabolism [34]. Increased ROS can damage the structure and function of bacterial biomolecules, including proteins, nucleic acids, and lipids, and can exert antibacterial effects [35]. Tandem mass tag quantitative proteomics and non-targeted metabolomics were used to investigate the inhibitory effect and related mechanisms of anthocyanins from *Aronia melanocarpa* on *Escherichia coli* O157:H7. The results showed that proteins involved in the glycolysis pathway (*FbaA* and *GlpX*) were upregulated after treatment with *Aronia melanocarpa* anthocyanins at 0.313 mg/mL, indicating disruption of *Escherichia coli* energy metabolism [36]. Anthocyanins from *bougainvillea* flowers (*Bougainvillea spectabilis*), chili pepper fruit (*Capsicum annuum*), and pomegranate peel (*Punica granatum*) inhibited Gram-positive and Gram-negative bacterial growth. The minimum inhibitory concentration of pomegranate peel anthocyanins was 1 μg/mL, while the others were 5 μg/mL [37]. The antibacterial characteristics corresponding to specific anthocyanins need to be experimentally determined.

Anthocyanins exert antibacterial effects through specific structural interactions with microorganisms. Anthocyanin hydroxyl groups react with lipids in microbial cell membranes through lipid peroxidation, causing membrane damage. In addition, under acidic conditions, positively charged anthocyanins interact with bacterial proteins and cell wall polysaccharides, thereby disrupting the bacterial life cycle. The conjugated aromatic rings in anthocyanins may also interact with the electron transport chain within microbial cells, affecting microbial metabolism and proliferation [38].

### 5.3. Other Biological Activities of Anthocyanins

In addition to antioxidant and antibacterial activities, anthocyanins also exhibit various biological activities, such as suppressing obesity, anti-cancer activity, nerve protection, retinal protection, immune response balancing, anti-aging activity, and anti-atherosclerosis [39].

## 6. Application of Anthocyanins in Food Preservation

Anthocyanins were widely available, and possessed excellent antioxidant and antibacterial activities. Anthocyanins exhibited good preservation effects on various foods, including fruits and vegetables, meats, aquatic products, and processed foods.

### 6.1. Application of Anthocyanins in Fruit and Vegetable Preservation

Research results showed that anthocyanins effectively delayed oxidation in fruits and vegetables, inhibited pathogen growth, reduced water loss, and maintained firmness, thereby preserving freshness (Table 2).

Anthocyanins had excellent preservative effects on fruits and vegetables such as blueberries, grapes, bananas, strawberries, apples, kiwifruit, and mushrooms. Polysaccharide-based packaging films were prepared using anthocyanins (purity, 25%) derived from solanum tubers, hydroxypropyl guar, and cellulose nanocrystals for the preservation of grapes and blueberries. The films inhibited *Staphylococcus aureus* activity in an in vitro experiment. Adding anthocyanins to composite films reduced moisture loss and oxidative spoilage in the fruits. In the experimental group, the shelf lives of blueberries and grapes extended to 12 and 20 days, respectively, at 25 °C and 65% relative humidity. The group without anthocyanins showed browning in grapes and more severe wrinkling in blueberries [40]. Multifunctional smart films made from a composite of black rice anthocyanins, chitosan, and *Phyllanthus emblica* L. polysaccharides extended blueberry shelf life from 6 days to 12 days at 4 °C, with a weight loss rate of only 5.68% compared with a chitosan film without active substances. The composite film effectively maintained fruit firmness and inhibited fruit browning [41]. Researchers prepared smart packaging films using anthocyanins, zeolitic imidazolate framework-8, carboxymethyl cellulose, and polyvinyl alcohol for grape preservation and freshness indication. Anthocyanins slightly enhanced the antibacterial activity of zeolitic imidazolate framework-8 in an in vitro experiment. The pH-responsive properties of anthocyanins enabled real-time indication of grape freshness. Experimental data showed that grapes in the zeolitic imidazolate framework-8 group with added anthocyanins lost less weight on the 7th day than those in the zeolitic imidazolate framework-8 group. Furthermore, adding anthocyanins significantly improved sensory evaluation indicators for grape appearance, texture, aroma, and color [42]. Bilayer films were prepared using red radish extract, agar, TiO_2_, and neem essential oil to preserve bananas. Anthocyanin content in red radish extract was 364.0 ± 5.8 mg/g. Red radish extract enhanced the antioxidant and in vitro antibacterial properties of the films in chemical experiments. The prepared films containing red radish extract suppressed banana browning and weight loss, inhibited mold and yeast growth, and slowed firmness loss [43]. The pH-responsive fiber films for strawberry preservation and freshness indication were composed of blueberry anthocyanin extract, oxidized sodium alginate, nisin, and carboxymethyl chitosan. The anthocyanin content in the blueberry anthocyanin extract was 40%. Anthocyanins were the main chromogenic substances with pH-responsive characteristics. The composite fiber films containing nisin and anthocyanins inhibited hardness decline, weight loss, and decay in strawberries. The fiber films’ color changed with strawberry freshness. Furthermore, the fiber membrane’s color-response sensitivity to pH increased with increasing anthocyanin content [44].

In addition to whole fruits, anthocyanins also preserve fresh-cut fruits effectively. Fibrous films made using elderberry anthocyanins, konjac glucomannan, and polyvinylpyrrolidone exhibited excellent preservation effects on fresh-cut apples. The elderberry anthocyanin content was 10%. Elderberry anthocyanins endowed the fibrous films with superior antioxidant properties. In chemical experiments, the films’ ABTS and 2,2-Diphenyl-1-picrylhydrazylradical free radical scavenging rates were 96.18% and 74.69%, respectively. The composite fibrous films inhibited browning, weight loss, and hardness decline, and increased total soluble solids in fresh-cut apples [45]. Functional fiber membranes were prepared using blueberry anthocyanins, 1-methylcyclopropene, acetylated starch, and pullulan to preserve and indicate the freshness of fresh-cut kiwifruit. Blueberry anthocyanins were extracted from blueberry pomace, and the anthocyanin content was 25%. The anthocyanins bound to 1-MCP-cyclodextrin inclusion complexes via hydrogen bonds and molecular interaction networks, enhancing the material’s mechanical properties. The functional fiber membrane extended the shelf life of fresh-cut kiwifruit from 3 to 5 days compared with the untreated group [46].

Anthocyanins can also be very effective in preserving vegetables. Biodegradable active packaging films were prepared using blueberry anthocyanins and soy protein isolate to preserve edible mushrooms (*hypsizygus marmoreus*). Anthocyanins bound to soy protein isolate via hydrogen bonds. The anthocyanins improved film properties, including oxidation resistance and water vapor barrier performance, in chemical experiments. Anthocyanins in composite films reduced weight loss, respiration rate, and browning; decreased malondialdehyde (MDA) levels; and maintained the enzyme activities of superoxide dismutase (SOD) and catalase (CAT) in edible mushrooms [47].

### 6.2. Application of Anthocyanins in Meat Preservation

Meat is susceptible to spoilage during storage due to the growth of pathogenic microorganisms and the oxidation of lipids and proteins, shortening shelf life [48]. Therefore, the question of how to inhibit microbial growth and oxidative deterioration; maintain meat quality, flavor, and nutrition; and reduce food safety risks needs to be resolved. The core advantages of anthocyanins in meat preservation lie in reducing lipid oxidation and microbial contamination, as well as indicating freshness (Table 3).

Anthocyanins have been shown to effectively preserve pork. A bilayer film system was constructed to preserve pork. The inner indicator layer consisted of potato starch, k-carrageenan, ε-lysine, and cranberry anthocyanins, while the outer protective layer consisted of chitosan, gelatin, and ZnO-doped carbon dots. Cranberry anthocyanins were responsive to pH and ammonia. When pork was stored at 20 and 4 °C, the prepared films changed color from red to yellowish-green. This was because the longer the pork was stored, the higher the degree of spoilage, resulting in more total volatile base nitrogen (TVB-N), which caused pH changes and then led to structural changes in the anthocyanins, transforming them from the flavonoid cation structure to the chalcone structure [49]. Intelligent bilayer pH-sensitive pads were prepared using blueberry anthocyanins, citric acid, and yellow lake as a composite indicator for real-time indication of the freshness of chilled pork. Blueberry anthocyanins exhibited pH/NH_3_ responsiveness. The intelligent composite pads provided excellent color indication of pH/TVB-N in chilled pork. The pads appeared red when the pork was fresh, turned orange when it was semi-fresh, and turned green when it was spoiled [50]. We prepared composite films using saskatoon berry anthocyanins, carboxymethyl cellulose, and xylan. Saskatoon berry anthocyanins formed hydrogen bonds with the xylan/carboxymethyl cellulose matrix, improving the films’ mechanical properties, moisture resistance, thermal stability, hydrophobicity, and in vitro antibacterial activity. Saskatoon berry anthocyanins also enhanced the films’ antibacterial properties in a pork-preservation application. The addition of anthocyanins to films extended the shelf life of pork by at least 2 days at 4 °C [51]. *Aronia melanocarpa* anthocyanins effectively inhibited *S. aureus and E. coli growth* in a chemical experiment. In a pork preservation experiment, *Aronia melanocarpa* anthocyanins suppressed microbial growth. *Aronia melanocarpa* anthocyanins also delayed color and odor deterioration in the pork and reduced moisture loss. Compared with sodium alginate/pectin films, films with added anthocyanins were sensitive to ammonia and could indicate pork freshness during storage. The films were red when the pork was fresh, became colorless when it was near-fresh, and turned purple after spoilage [52].

In addition to pork, anthocyanins can also be used to preserve chicken. Researchers prepared smart packaging films using grape skin anthocyanins, betalains, shikonin, and a gelling gel. To improve stability, the anthocyanins were chemically modified with mandelic acid. The smart films containing modified anthocyanins inhibited increases in pH, TVB-N, and thiobarbituric acid (TBARS) in chicken breast during storage compared with films containing unmodified anthocyanins. However, compared with films containing modified anthocyanins, the composite films containing un-modified anthocyanins had more noticeable color changes, indicating the freshness of chicken breast stored at 4 °C through color changes, from purplish-red (0 days) to purple (3 days), to dark purple (6 days), to yellowish-green (9 days), and finally to light yellow (12 days) [53]. Researchers prepared composite films using *Syzygium cumini* anthocyanin extract, chitosan, 2-hydroxyethyl methacrylate, methacrylic acid, and carbon quantum dots to indicate chicken freshness. The pH responsiveness of *Syzygium cumini* anthocyanin extract was the basis for the color indication of the films. When chicken meat spoiled, the pH rose, and the composite film’s color changed from purplish-red (fresh) to brownish-green (spoiled) [54].

Anthocyanins also exhibited excellent preservative effects on other meats. Researchers prepared a composite spray using black rice anthocyanins, egg yolk immunoglobulin, and fucoidan to preserve yak meat. Adding fucoidan effectively stabilized the anthocyanin-containing complex. The composite spray slowly released anthocyanins, thus exerting sustained antioxidant activity. Preservation results showed that compared with the anthocyanin spray, the composite spray inhibited the decline in yak meat firmness, elasticity, and chewiness and effectively maintained color stability. However, compare with the composite spray, anthocyanin inhibited pH increase of yak meat on day 9 at 4 °C [55]. Smart nanocomposite films were prepared using chitosan, κ-carrageenan, rutin nanoparticles, and grape skin anthocyanins. The anthocyanins exhibited antioxidant properties in a chemical experiment. The color changes of grape skin anthocyanins in response to pH and ammonia enabled the prepared smart films to indicate the freshness of fresh chicken and beef for 48 h at 25 °C. As pH and TVB-N increased during storage, the color of the anthocyanin- and rutin-containing composite films changed from purple to green. The composite films inhibited increases in total viable count (TVC), peroxide value, pH, and TVB-N, thereby extending beef shelf life [56]. Smart packaging films were prepared using purple sweet potato pigment (anthocyanin content, 100–120 mg/g) microcapsules (loading efficiency, 80.33 ± 0.63%), nanosilver, gellan gum, and sodium alginate to indicate the freshness of chilled mutton. The color of the indicator membrane containing anthocyanins changed in an ammonia environment according to the freshness of the chilled mutton, successively showing purplish-red (fresh meat), grayish-green (sub-fresh meat), and dark blue (rotten meat) [57].

### 6.3. Application of Anthocyanins in the Preservation of Aquatic Products

Aquatic products were prone to spoilage due to lipid oxidation, protein decomposition, and microbial proliferation [58]. Anthocyanins slowed the quality deterioration rate, maintained the nutritional value, and extended the shelf life of aquatic products (Table 4). During aquatic product spoilage, alkaline substances were produced. They altered the structure and color of anthocyanins, which indicated the freshness of aquatic products.

Anthocyanins can be used to preserve shrimp. Slow-release active smart films prepared using phenolic acid, *Aronia melanocarpa* anthocyanins, and microencapsulated carvacrol extended the shelf life of shrimp (*Litopenaeus vannamei)* by 12 h. As shrimp deteriorated from fresh to spoiled, the shrimp muscle protein decomposed, nitrogenous compounds were released, and the pH increased. Because anthocyanins are highly pH-responsive, the color of the composite smart films changed from pink to blue, providing a visual indication of freshness [59]. Nanocomposite films were prepared using chitosan, corn starch, and *Vaccinium vitis-idaea* anthocyanin extract for shrimp preservation. The anthocyanin extract enhanced the antioxidant activity of the films and their ability to inhibit *S. aureus* and *E. coli* in in an in vitro experiment. Films containing anthocyanin extract inhibited increases in pH, weight loss, TVB-N production, lipid oxidation, and microbial growth and reproduction in shrimp at 4 °C, maintaining shrimp freshness for at least 2 days. The nanocomplexes were prepared using whey protein isolate, chitosan hydrochloride, carboxymethyl chitosan, and anthocyanin extract. Compared with free anthocyanins, the embedding method delayed anthocyanin release, making the films’ antioxidant effect more durable in a chemical experiment and their preservation effect in food stronger [60]. Films were prepared using chitosan, gelatin, and blue honeysuckle berry anthocyanin extracts for the preservation and freshness indication of shrimp. Anthocyanins exhibited free radical scavenging and antibacterial activity, as well as sensitivity to pH and ammonia in a chemical experiment. Compared with no film, these composite films suppressed increases in TVB-N and TVC in shrimp. As the shrimp transitioned from fresh to spoiled, the anthocyanin-containing films changed color from dark grayish-purple to dark brown. Furthermore, to slow anthocyanin release and increase stability, blue honeysuckle berry anthocyanins were loaded onto esterified porous starch to form composite particles containing solidified anthocyanins. However, TVB-N and TVC values indicated that this processing method had limited effectiveness in enhancing freshness preservation [61]. A biohybrid material formed by loading jambolan fruit anthocyanin extract onto montmorillonite was used to indicate shrimp freshness. The presence of anthocyanins allowed this material to change color from pink (fresh) to blue (spoiled) at 25 °C [62]. Intelligent packaging films were prepared using chitosan, cellulose nanocrystals, and blueberry anthocyanins (purity, 25%) for delaying spoilage and indicating the freshness of shrimp. Blueberry anthocyanins changed color by responding to pH. The composite film limited the TVB-N content of shrimp at 4 °C [63]. The film containing rosella extract (anthocyanin content: 407.22 μg/g) showed excellent pH responsiveness and indicated shrimp spoilage through color changes. The color of the film turned from purple when the shrimp were fresh to dark blue when they were spoiled. At the same time, rosella anthocyanins also conferred antioxidant properties to the film in a chemical experiment. This composite film group extended shrimp shelf life by at least 2 days at 4 °C compared with the unpackaged group. The stability of rosella anthocyanins was enhanced after they were partially embedded in carboxymethyl chitosan and chitosan hydrochloride. Films containing partially encapsulated anthocyanins extended shrimp shelf life by at least 1 day compared with films containing non-encapsulated anthocyanins; however, partial encapsulation weakened the film’s color indication of shrimp freshness [64]. Green intelligent fresh-keeping films were prepared using chitosan, polysaccharides from *Phyllanthus emblica* L., and black rice anthocyanins. In a chemical experiment, anthocyanins enhanced the antioxidant capacity and antibacterial activity of these films. Compared with chitosan films, films containing anthocyanins and *Phyllanthus emblica* L. polysaccharides inhibited TVB-N production, microbial growth and reproduction, lipid peroxidation, reductions in myofibrillar protein content, and decreases in Ca^2+^-ATPase activity in shrimp. Independent of the preservation experiment, the authors conducted a real-time freshness indication experiment on shrimp. The results showed that after the shrimp were placed at 25 °C for 14 h, the composite intelligent films changed from red to brown, effectively indicating the freshness of the shrimp [41]. Active intelligent packaging films were prepared using carrageenan, gelatin, licorice root-derived carbon dots, silica nanoparticles, and red cabbage anthocyanin extract (content, 184 mg/100 g). The addition of anthocyanin extract enhanced the antioxidant and antibacterial activities of the films and endowed them with pH-responsive color properties in a chemical experiment. After shrimp were stored at 28 °C for 24 h, the films containing anthocyanins changed in color from pink to green. Red cabbage anthocyanin extract inhibited the increases in pH, redness, and TVC of shrimp [65].

Besides shrimp, anthocyanins can be well applied to fish preservation. Intelligent films were prepared using blueberry anthocyanins, carboxymethyl cellulose sodium, xanthan gum, and gallic acid to preserve sturgeon. The results showed that blueberry anthocyanins exhibited in vitro antibacterial, antioxidant, pH-sensitive, and ammonia-sensitive properties in chemical experiments. The prepared films inhibited increases in TVB-N and TVC in sturgeon and delayed spoilage. As the sturgeon changed from fresh to spoiled, the smart films’ color shifted from purplish-red to colorless or light yellow [66]. Researchers prepared nanocomposite films using black rice bran anthocyanins, oxidized chitin nanocrystals, and gelatin to indicate hairtail freshness. In a chemical experiment, black rice anthocyanins enhanced the films’ antioxidant properties. TVB-N in hairtail was inhibited by black rice bran anthocyanins. As the hairtail changed from fresh to spoiled, the color of the films containing black rice bran anthocyanins shifted from purple to brown [67]. Researchers prepared multifunctional films using corn starch, polyvinyl alcohol, and blueberry anthocyanins (cyanidin cation, 25 wt%) for preserving and indicating salmon freshness. The blueberry anthocyanins showed excellent pH responsiveness. As salmon transitioned from fresh to spoiled, the smart composite film’s color shifted from purplish-red to yellow-green. The prepared films extended salmon shelf life by 1.5 days at 4 °C. Furthermore, anthocyanins were loaded onto ovalbumin–sodium alginate to form a nanocomposite. Plasma-modified corn starch, polyvinyl alcohol, and the nanocomposite were used to prepare smart films. This film featured a network structure that effectively delayed anthocyanin release and degradation [68]. Composite films prepared from *Gelidium amansii* polysaccharides and lingonberry anthocyanins were used to preserve and indicate the freshness of crucian carps. Lingonberry anthocyanins exhibited pH and ammonia responsiveness and significantly enhanced the antioxidant activity, in vitro antimicrobial activity, and oxygen barrier properties of the films in chemical experiments. Adding lingonberry anthocyanins to films inhibited increases in pH, TVB-N, and TVC values, extending the fish’s shelf life to two weeks at 4 °C. As the fish transitioned from fresh to spoiled, the composite film’s color shifted from pink to brownish-purple [69]. Researchers prepared active films using polyvinyl alcohol, fish gelatin, and *Solanum nigrum* L. anthocyanins to preserve and indicate the freshness of grass carp. Because of the anthocyanins, the active films were responsive to pH and NH_3_. The films exhibited antioxidant and in vitro antimicrobial activities in chemical experiments. Adding anthocyanins to films effectively preserved grass carp. During storage of grass carp at 4 °C, the films changed from dark pink to colorless as the fish transitioned from fresh to spoile [70].

In addition to fish and shrimp, anthocyanins can also be applied to crabs. Bilayer films consisting of an oxygen barrier layer and a sensing layer were used to indicate the freshness of Chinese mitten crabs. The sensing layer of the films was composed of mulberry anthocyanins and gellan gum. Mulberry anthocyanins exhibit favorable responsiveness to pH. During crab spoilage, trimethylamine content increased, raising the pH level. As the crab transitioned from fresh to spoiled, the film changed color from pink to dark green [71].

### 6.4. Application of Anthocyanins in the Preservation of Processed Foods

Preserving processed foods requires inhibiting microbial growth, delaying oxidative deterioration and moisture loss, maintaining original quality, flavor, and nutritional content, and ensuring food safety for consumption. Anthocyanins exhibited excellent preservation effects on processed foods (Table 5). The study results showed that adding camphor fruit anthocyanins to fresh tofu inhibited spoilage at 30 °C [72]. Researchers prepared intelligent films using purple cabbage anthocyanins, curcumin, pullulan, polyvinyl alcohol, and soybean shell nanocellulose to preserve dried tofu and indicate its freshness. anthocyanins and curcumin both showed pH-responsive and in vitro antibacterial properties. The intelligent films containing anthocyanins and curcumin suppressed browning and increases in pH, TNB-N, and TVC in dried tofu, and extended its shelf life from 8 days to 12 days at 4 °C. As the dried tofu changed from fresh to spoiled, the composite films’ color changed from deep red to deep purple [73]. Intelligent labels were prepared using cellulose acetate, red cabbage anthocyanin extract, and thymol to preserve tomato sauce and indicate its freshness. Red cabbage anthocyanin extract was responsive to pH and NH_3_. Thymol and the anthocyanins both exhibited antioxidant activity in a chemical experiment. As the tomato sauce changed from fresh to spoiled, the label color shifted from purple to green, then to yellow. The label inhibited the decrease in pH and the color change of tomato sauce [74]. Multifunctional double-layer films were prepared for cheese preservation. The inner film was prepared using elderberry anthocyanins and konjac glucomannan, and the outer film was prepared using β-cyclodextrin, oregano essential oil, and chitosan. Elderberry anthocyanins exhibited pH responsiveness and antioxidant and in vitro antibacterial activities in chemical experiments. Adding elderberry anthocyanins to the double-layer films inhibited darkening of cheese color, increased pH and hardness, and weight loss. Furthermore, compared with the composite film containing elderberry anthocyanins and konjac glucomannan, the double-layered film containing β-cyclodextrin, essential oil, and chitosan showed better preservation of cheese [75]. Researchers prepared active intelligent packaging films using *Rhododendron pulchrum Sweet* anthocyanins, carrageenan, polyvinyl alcohol, and ε-polylysine for preservation and freshness indication of Chinese pork meatballs. The anthocyanins showed pH responsiveness, ammonia sensitivity, antioxidant activity, and in vitro antibacterial properties in chemical experiments. The prepared composite films containing anthocyanins and 6% ε-polylysine inhibited weight loss, pH increase, color change, lipid oxidation, TVB-N and TVC increase, taste change, and sensory quality decline of simmered Chinese pork meatballs [76].

## 7. Summary and Perspective

In this article, we reviewed the sources, extraction methods, antioxidant and antibacterial mechanisms, and applications of anthocyanins in food preservation. Anthocyanins have been shown to be promising materials for application in food preservation. Table 2, Table 3, Table 4 and Table 5 show that the composite films containing anthocyanins can extend the shelf life of food, provide visible color changes based on the food freshness state, or both.

Anthocyanin sources can be categorized as vegetable, fruit, grain, flower, and other sources. Anthocyanin-related materials from different sources may differ in antibacterial activity and antioxidant capacity, but no consistent ranking between sources has yet been established. Anthocyanins were incorporated into films either in purified form or as anthocyanin-containing extracts. These extracts may also contain phenolic compounds or other active compounds. Regarding antioxidant activity, the pomegranate peel extract showed the highest activity, followed by the *bougainvillea* flower extract and the chili pepper fruit extract. The anthocyanins detected in pomegranate peel extract and chili pepper fruit extract were delphinidin-3-O-glucoside, pelargonidin-3-O-glucoside, and cyanidin-3-O-glucoside, whereas *bougainvillea* flower extract also contained peonidin-3-O-glucoside. The cyanidin-3-O-glucoside contents of pomegranate peel, chili pepper fruit, and *bougainvillea* flower extracts were 9.94, 14.36, and 10.06 μg/g, respectively, whereas their total phenolic contents were 466, 14, and 180 mg GAE/g, respectively. The study found a linear relationship between the antioxidant activity of these three extracts and their total phenolic content [37]. In an assay assessing the inhibition of *Bacillus cereus*, anthocyanin fractions from black chokeberry (1.6 μg/mL) and blackcurrant (0.3 μg/mL) showed significant antibacterial activity, whereas anthocyanin fractions from elderberry (3.9 μg/mL) did not exhibit significant inhibitory effects under the conditions tested. The major anthocyanins in all three groups were cyanidin-3-O-galactoside, cyanidin-3-O-sambubioside, and cyanidin-3-O-rutinoside, accounting for approximately 70–80% of the total anthocyanin content [77]. These results suggested that the biological activity of anthocyanin extracts was related not only to the types and contents of anthocyanins but also to other phenolic compounds present in the extracts. The antibacterial and antioxidant activities of anthocyanins help delay food spoilage.

Based on human studies and animal toxicological experiments, toxic effects of anthocyanin extracts have not been found. In one human study, 34 patients with ulcerative colitis were randomly divided into two groups. The experimental group received 800-1000 mg of bilberry extract orally three times daily (anthocyanin purity 36%) for 8 weeks. Fecal calprotectin decreased significantly from baseline in the anthocyanin extract group, whereas it remained essentially unchanged from baseline in the placebo group. No differences were observed in Mayo score, rectal bleeding, or stool frequency. No adverse reactions were observed during the study [78]. In another human study, 61 healthy older adults were randomly divided into two groups and consumed a freeze-dried blueberry powder beverage containing 302 mg anthocyanins or a placebo daily for 12 weeks. The blueberry group showed improvements in fecal calprotectin and 24 h systolic blood pressure, and increased accuracy in immediate word recall and the task-switching task. Urinary (poly) phenol excretion in the blueberry group increased by 1235 μmol compared with the placebo group. No differences were observed in arterial stiffness, 24 h ambulatory diastolic blood pressure, office blood pressure, cerebral blood flow, blood lipids, other cognitive tasks and mood scores, fasting plasma total (poly)phenols metabolites, or gut microbiota. No adverse reactions were observed during the study [79]. Eight-week-old Sprague Dawley rats were used and dosed by oral gavage. In the acute toxicity test, a 300 mg/kg dose group was established and dosed by gavage for 14 days. In the subacute toxicity test, doses of 30, 15, and 7.5 mg/kg were administered by continuous gavage for 28 days. In the acute and subacute toxicity tests, no differences were observed in organ weights, hematology, liver and kidney function, or histopathology compared with the respective control groups [80]. Under the European Union regulations, when anthocyanins are used as a food additive, their permitted sources are fruits and vegetables, mainly grape skin and blackcurrant. Based on toxicological and general exposure assessments of anthocyanins from aqueous extracts of grape skin and blackcurrant, the European Union considers that anthocyanins derived from water extracts of grape skin and blackcurrant are unlikely to raise safety concerns. The Joint FAO/WHO Expert Committee on Food Additives has set an acceptable daily intake of 2.5 mg/kg bw/d for grape skin anthocyanins. In food processing, anthocyanins may be used as a food additive in foods such as ripened cheese; canned or bottled fruit and vegetables; fruit or vegetable spreads; casings and coatings; and decorations for meat, protein products, noodles, batters, etc [81]. For most foods, anthocyanins are added quantum satis (as required), while maximum limits are set for a small number of foods. For example, the limit for anthocyanins in fruit-flavored breakfast cereals is 200 mg/kg [81,82]. During food preservation, some prepared anthocyanin-containing active films came into contact with the food. During this process, the film components may migrate into the food. Some materials used anthocyanin extracts containing other components, TiO_2_, ZnO/carbon dots, or other nanoparticles. The safety and migration of these ingredients should be carefully considered. The characteristics of nanoparticles include high reactivity and large surface area. When cells or tissues are exposed to nanoparticles, they may interact with each other, leading to potential toxicological lesions [83]. Many studies have extracted anthocyanins from plant materials and obtained anthocyanin extracts of different concentrations. However, in addition to anthocyanins, the extract may also contain additional phenolic compounds and other components. This makes the safety of extracts containing anthocyanins uncertain. The European Union permits anthocyanins for use as food additive [81]. But no regulations worldwide currently target anthocyanin-based smart packaging materials. The European Union regulations on food-contact materials stipulate that such materials must not alter the taste, color, aroma, or other properties of food and must not allow substances hazardous to health to migrate into food [84]. Currently, migration and toxicological data for anthocyanin-containing preservative films are incomplete; therefore, when anthocyanin extracts are used in food-contact materials, separate safety assessments of migration and toxicology for anthocyanin extract-containing preservative films are required.

Anthocyanins are easily degraded by various factors such as light, temperature, oxygen, and pH, which limits their application in food preservation [85]. Under acidic conditions, the degradation rate of anthocyanin extracts after heating was relatively low. At 60 or 70 °C, the degradation rate of anthocyanin extracts from fruits, vegetables, and flowers was less than 20%. However, heating under neutral and alkaline conditions increased anthocyanin instability and significantly increased their degradation rate [86]. Exposure of anthocyanins to light triggers degradation. When blueberry anthocyanins were exposed to ultraviolet radiation (10 W) for less than 24 h, their degradation was not significant. However, as time increased, especially after 96 h, the degradation rate of blueberry anthocyanins accelerated significantly. The longer the UV irradiation time, the higher the first-order kinetic reaction rate of blueberry anthocyanins [21]. The degradation of blueberry anthocyanins increased with increasing pH. Under alkaline conditions, ultraviolet-light-induced degradation of blueberry anthocyanins was faster [21]. Many kinds of foods experienced an increase in pH during storage. As food storage time extended beyond the initial period, anthocyanins were expected to play a preservation role. However, the increase in pH led to the degradation of anthocyanins, usually resulting in a decrease in activity, which restricted the application of anthocyanins in food preservation. The longer the exposure time of anthocyanins to oxygen, the higher the decomposition rate. For example, after 60 days of oxygen exposure, the retention rate of anthocyanins in red cabbage was 53.24 ± 0.03%. After 90 days of exposure, the retention rate of anthocyanins significantly decreased to less than half (47.43 ± 0.03%) [87]. According to a reviewed study, at 60 °C, the average half-life of anthocyanin degradation is about 19.7 h in fruits and 22.28 h in vegetables. However, increasing the temperature shortens these half-lives [86].

The test data indicated that anthocyanin-containing preservative films can effectively protect food’s sensory quality. For example, compared with the anthocyanin-free group, the double-layer pad containing anthocyanin group delayed the texture deterioration of chilled pork. At the end of storage, the hardness of these two groups decreased from 307.50 to 183.06 g and 207.15 g, respectively, and the springiness decreased from 0.99 to 0.43 and 0.57, respectively [50]. In addition, a comparison of the anthocyanin-containing group with the anthocyanin-free group showed that at 15 d, springiness decreased from 7.19 to 5.54 and 5.45, respectively; hardness decreased from 22.75 and 22.65 to 13.83 and 12.06, respectively; adhesiveness increased by 46.3% and 53.7%; and the sensory score of the anthocyanin-containing group on day 12 was still higher than that of the anthocyanin-free group [52]. However, excessive migration can compromise food sensory quality. Anthocyanins migrating into food may cause food discoloration and affect sensory quality [88].

Researchers have studied anthocyanin-containing composite films for food preservation, but no such products are currently on the market. To move anthocyanin-containing cling films from the laboratory to industrial-scale production, manufacturing standards are needed. However, no unified global standard exists for producing anthocyanin-containing cling films. Therefore, to achieve large-scale production, reasonable specifications for standardized production need to be formulated. Regulations governing anthocyanins as food additives exist worldwide; however, no single approval or regulatory framework specifically addresses anthocyanin-based composite films as a product category. People have relatively acceptable sensory acceptance of anthocyanin composite preservation films. In a comparison of sensory scores on a 9-point scale, the 0.75% black rice anthocyanin film group showed an overall preference of 5.60 after 20 days of thermal oxidation, higher than the control group without anthocyanins (score of 2). After 20 days of UV oxidation, the overall preference for anthocyanin-containing cling film was 5.50, while the control group had a preference of 2.0 [89]. In another study, after the film was stored at 38 °C for 6 months, the overall acceptance score of the film containing 5% grape skin anthocyanins was 5.64, which was higher than that of the membrane without anthocyanins (score of 4.92) [90]. The film-forming substances of composite films may detach owing to polymer swelling and matrix degradation, resulting in unintended release and subsequent migration into food [91]. High production cost is one obstacle to the commercialization of anthocyanin preservative films [92]. Traditionally, anthocyanins are extracted from fruits; industrially, agricultural by-products (e.g., fruit pomace and peel residue) can be used as anthocyanin sources [78,93]. With technological development, the price of high-purity anthocyanins has continued to decline [11]. However, the matrix materials used to form films, other than anthocyanins, also incur certain costs. Anthocyanins contain many unstable phenolic hydroxyl groups and unsaturated bonds, making them prone to oxidation, hydrolysis, and structural transformation. Therefore, they are sensitive to environmental factors such as pH, temperature, light, oxygen, enzymes, and metal ions, and readily degrade [94]. Anthocyanin-containing composite films exhibit poor long-term stability. In the laboratory, techniques such as encapsulation, co-pigmentation, and acylation modification have been used to treat anthocyanin composite films [95]. Under certain conditions, these methods can stabilize the properties of anthocyanin composite films; however, no conclusive evidence exists on their long-term stability, and further investigation is needed.

During composite film preparation, anthocyanins mainly interacted non-covalently with the film substrates, primarily through hydrogen bonds, and electrostatic repulsion also occurred. During food preservation, the composite films maintained their antioxidant/antibacterial activity, similar to their effects in chemical tests. For example, blueberry anthocyanin significantly increased radical scavenging activity in chemical experiments and significantly increased SOD and CAT activities in mushrooms [47]. In chemical experiments, saskatoon berry anthocyanins showed antibacterial activity, and in food applications, they also significantly inhibited TVC in pork [51]. Red cabbage anthocyanin showed an antibacterial effect in chemical experiments and inhibited TVC in shrimp [65]. A composite film containing *Rhododendron pulchrum Sweet* anthocyanins inhibited microorganisms in chemical tests and inhibited TVC in simmered Chinese pork meatballs [76]. In food applications, the release and migration of anthocyanins in food should be fully considered. In the experiment simulating the release of anthocyanins into food, the release rates of anthocyanins in distilled water, 10% ethanol, 50% ethanol, and 95% ethanol differed between plasma-treated corn starch/polyvinyl alcohol films containing free anthocyanins and plasma-treated corn starch/polyvinyl alcohol films containing anthocyanins -loaded nanocomposite suspension. In distilled water, anthocyanin release rates in these two groups were 74.56% vs. 23.14%; in 10% ethanol, 59.64% vs. 18.2%; in 50% ethanol, 43.01% vs. 11.64%; and in 95% ethanol, 16.94% vs. 4.77%. Encapsulation reduced the anthocyanin release rate, and increasing ethanol concentration further decreased release. Possible reasons included hydrogen bonding, electrostatic interactions, and a Ca^2+^-mediated “egg-box” structure. Low water content was not conducive to the dissolution of the hydrophilic starch/polyvinyl alcohol matrix [68]. In water, 50% ethanol, and 95% ethanol, total phenol release from the composite film increased, possibly due to the poor water solubility of the benzene ring structure of flavonoids in *Solanum nigrum* L. anthocyanins [70]. Generally, the preservation effect of anthocyanins increased with anthocyanin loading. For example, higher saskatoon berry anthocyanin loading (0.1, 0.15, 0.2, 0.25 wt%) in the composite films led to stronger antibacterial and pH-inhibiting effects [51]. As the black rice bran anthocyanin content increased (0, 20, 40, 80, and 100 mg) in the composite films, the composite film’s inhibitory effect on TVB-N increased [67]. As the content of *Solanum nigrum* L. anthocyanins in the composite film increased (0, 0.2%, 0.3%), the inhibitory effect of the film on TVB-N and TVC became stronger [70]. However, some studies reported different results. For example, the loading amounts of purple potato anthocyanin microcapsules in the composite membrane were 1, 2, 3, and 4 g. However, the most significant color change in the membrane was observed in the group containing 2 g of purple potato anthocyanin microcapsules [57]. Anthocyanin instability may increase when they respond to pH changes and change color. In practical applications, many studies store food at low temperatures (such as 4 °C). At low temperatures, anthocyanin stability improves. A Study has shown that low-temperature environments can cause a greater ΔE in the color of anthocyanins [96]. Therefore, a low temperature may alleviate the contradiction between color change and stability of anthocyanin films or labels.

We compared anthocyanin sources with those in previous review papers on anthocyanin preservation. Kalita et al. [97] reported common anthocyanins such as blueberry anthocyanins and red cabbage anthocyanins. We reported some other sources of anthocyanins, such as cranberry anthocyanins, saskatoon berry anthocyanins, *Syzygium cumini* anthocyanins, blue honeysuckle berry anthocyanins, black rice bran anthocyanins, lingonberry anthocyanins, *Solanum nigrum* L. anthocyanins, camphor fruit anthocyanins, and *Rhododendron pulchrum Sweet* anthocyanins. Most of these new anthocyanin sources come from plant fruits, with a small number from flowers and agricultural by-products, indicating that these sources have received recent attention. Our content further enriched the sources anthocyanins, providing a broader reference for the application of intelligent active preservation films. This review and Shi et al. [11] reported classic anthocyanin extraction methods, such as solvent extraction, ultrasound-assisted extraction, and microwave-assisted extraction. Shi et al. [11] and Tan et al. [98] reported anthocyanin extraction yields generally ranging from 2.9 to 6.1 mg/g. However, we found that (Table 1) the extraction yields of anthocyanins using deep eutectic solvent-assisted ultrasound extraction and microwave-assisted deep eutectic solvent extraction were higher, at 16.63 and 6.442 ± 0.135 mg/g, respectively [26,28]. This indicates that deep eutectic solvents offer significant advantages for anthocyanin extraction. Our content incorporated the latest anthocyanin extraction methods, reflecting advances in extraction techniques. Anthocyanins are easily degraded by light, temperature, oxygen, and pH [85]. Ma et al. [99] reported that microencapsulation and adding auxiliary colors were the primary methods for stabilizing anthocyanins. Here, we summarized methods such as encapsulation, acylation, co-pigmentation, improving the structure of packaging films, biohybrid approaches, and chemical modification that have been used to inhibit the degradation of anthocyanins [53,59,62,68,100,101]. However, these methods are not universal solutions to anthocyanin instability. The actual stability effect when using these methods depends on the specific formula. For example, research has shown that compared with natural blueberry anthocyanins (purity, 368.4 mg/g), acylated anthocyanins have lower retention rates at low acidity (pH ≤ 3), indicating lower pH stability. The retention rates of natural anthocyanins and acylated anthocyanins at pH = 2 were approximately 85% and 70% at the 50th min [102]. Kalita et al. [97] summarized applications of intelligent films and labels containing anthocyanins on fruits and vegetables, milk and dairy products, and muscle foods. We have basically covered these contents as well. In addition, we added discussions of processed foods such as tofu, dried tofu, tomato sauce, and simmered Chinese pork meatballs. We expanded the applications of anthocyanin-based smart films and labels. From the perspective of extending shelf life and ensuring food safety, this may help promote the development of the processed food industry. The sensing mechanism of anthocyanins for color changes is typically in response to pH and ammonia, which causes the color of the film to change, as also reported by Kalita et al. [97]. Some methods were used to increase the color difference, making it easier to recognize. For example, the film composed of anthocyanins, xanthan gum, gallic acid, and sodium carboxymethylcellulose had a larger ΔE than the film only composed of anthocyanins and sodium carboxymethylcellulose, and the color of these two composite films changed from purplish-red to light yellow and yellowish-green, respectively, after fish were stored at 4 °C for 10 days [66]. We have summarized the preservation effect of intelligent composite cling films containing anthocyanins on foods, which is also reported by Shi et al. [11]. In addition, we focused on data on extending food shelf life. Extending food shelf life helps reduce food waste. According to the extended shelf-life data, the preservation effect of composite films containing anthocyanins is more intuitively demonstrated. Regarding technological limitations, Kalita et al. [97] discussed possible solutions to anthocyanin instability, the challenges of film stability testing from laboratory to practical applications, and recommendations for regulatory approval of films. In addition, ensuring anthocyanin quality stability is quite difficult. Because of the influence of picking time, region, and other factors, it is difficult to ensure that each batch has identical properties, even when sourced from the same plant.

Currently, the main focus is on preparing active intelligent films with anthocyanins. To further preserve food and reduce food waste, anthocyanin-based preservation can be combined with other technologies, such as air-modulated storage, vacuum packaging, and low-temperature refrigeration. In addition, future work should optimize the amount of anthocyanins added, improve the addition process, and develop low-cost, more effective preparation methods.

## Figures and Tables

**Table 1:** Sources, extraction methods, and extraction yields of anthocyanins.

Sources of Anthocyanins	Extraction Methods	Extraction Yields	References
Fruit skin of *Rhodomyrtus Tomentosa* (Ait.) Hassk	Solvent extraction	4.358 ± 0.045 mg/g	[23]
*Clitoria ternatea* flowers	Solvent extraction	433.89 to 570.31 mg/100 g	[27]
Purple sweet potatoes	Ultrasound-assisted compound enzymatic extraction	2.27 mg/g	[25]
*Aronia melanocarpa*	Deep eutectic solvent-assisted ultrasound extraction	16.63 mg/g	[28]
*Purple-heart Radish*	Microwave-assisted deep eutectic solvent extraction	6.442 ± 0.135 mg/g	[26]

**Table 2:** Researches on the application of materials containing anthocyanins in fruits and vegetables preservation.

Food Category	Sources of Anthocyanins	Other Main Materials	Comparator	Freshness Indicator Response Factor	Color Changes	Main Results	Main Limitation	References
Grapes (12 g), Blueberries (2 g)	Solanum tubers anthocyanins, solanum tubers extract, purity 25%, loading 0.1 g.	Hydroxypropyl guar gum (HPG), cellulose nanocrystals (CNCs), and deep eutectic solvents	HPG/CNC vs. HPG/CNC/Anthocyanin	/	/	Suppress weight loss and stiffness decrease, the grapes were still relatively fresh on the 20th day, blueberries were still relatively fresh on the 12th day at 25 °C and 65% RH.	Many hydroxyl groups exist in polysaccharides, resulting in strong hygroscopicity of the film.	[40]
Blueberries	Black rice anthocyanins (BRA), black rice extract, 10% *w*/*w* based on chitosan (CS) dry weight.	CS, *Phyllanthus emblica* L. polysaccharides (PEP)	CS vs. CS-BRA-PEP-2	/	/	Maintain fruit firmness, inhibit browning, reduce weight loss on day 12 (11.89% vs. 5.68%), reduce malondialdehyde (MDA) level (*p* < 0.05), extend shelf-life by at least 6 days.	The current testing is mainly conducted in the laboratory. The antibacterial performance test only involves a portion of microorganisms. Long term stability optimization of artificial intelligence recognition systems is required.	[41]
Grapes	Anthocyanins (Ant), Ant (0.1%, *w*/*w*) was added to film-formation solution.	Carboxymethyl cellulose/polyvinyl alcohol (CP), zeolitic imidazolate framework-8 (ZIF-8)	ZIF-8/CP vs. Ant@ZIF-8/CP	pH	Yellow-brown to purple	Inhibit weight loss, improve the sensory evaluation indicators of grapes appearance, texture, aroma, and color, reduce weight loss on 7th day (approximately 8% vs. 6.05%), extend by at least 1 days	Compared with ZIF-8/CP film, the surface morphology of Ant@ZIF-8/CP film is rougher.	[42]
Banana	Red radish anthocyanins, red radish extract (RRE), purity 364.0 ± 5.8 mg/g.	Agar, TiO_2_, neem essential oil (NEO)	AG + AG vs. AG + AG-RRE	/	/	Suppress banana browning and weight loss, inhibit moulds and yeasts growth (2.33 ± 0.24 vs. 2.00 ± 0.12 log CFU/g), slow down firmness loss at 25 ± 1 °C.	Adding RRE resulted in a decrease in tensile strength and an increase in elongation-at-break of the film.	[43]
Strawberries	Blueberry anthocyanin extract (BA), purity 40%, loading 0.8% BA solution.	Oxidized sodium alginate (OSA), nisin, carboxymethyl chitosan (CMCS)	OSA2-CMCS vs. OSA2-N/A-CMCS (nisin and BA (N/A))	pH	Purplish pink to light yellow	Inhibit the hardness decline and weight loss, reduce decay by 40% after 5 days storage at room temperature.	Long-term stability was not evaluated in a real storage environment. They validated the film only on strawberries. Scaling up electrospinning and reducing costs remain challenges for industrial use.	[44]
Fresh-cut apples	Elderberry anthocyanins (EA), elderberry extract, purity 10%, EA (1 g) was added to 100 mL konjac glucomannan (KGM) solution.	KGM, polyvinylpyrrolidone (PVP)	PVP vs. KEA/PVP (KGM incorporated with EA (KEA))	/	/	Inhibit browning, inhibit weight loss after 72 h (27.43% vs. 21.13%), slow hardness decline after 72 h (2.53 vs. 2.91 N), extend shelf life by 24 h at 26 °C.	With the increase of KEA content, the tensile strength and elongation at break of KEA/PVP fiber film decrease.	[45]
Fresh-cut kiwifruit (130 ± 10 g)	Blueberry anthocyanins, blueberry pomace extract, purity 25%, 2% blueberry an-thocyanins was dissolved in the shell-layer spinning solution.	1-methylcyclopropene, acetylated starch, pullulan	Unprocessed vs. processed	pH	Light pink to dark purple	Inhibit the soluble solids content decrease, the conductivity increase, the total phenolic content increase, the ascorbic acid content decrease, the pH value increase, and color changes, extend shelf life from 3 days (untreated control) to 5 days at 25 °C (a 2-day extension).	/	[46]
Mushrooms (22 ± 3 g)	Blueberry anthocyanins (BA), 0.08% (*w*/*v* ratio) in the film-forming solution.	Soy protein isolate (SPI)	SPI vs. SPI/BA/0.08	/	/	Inhibit weight loss, browning, and respiration rate, achieve the lowest MDA content (1.49 mmol/kg), maintain higher superoxide dismutase (SOD) and catalase activities than the SPI film group during 8 days of storage at 4 ± 1 °C and 70 ± 2% RH, extend shelf life by 1 day.	Excessive BA reduces tensile strength and elongation at break, resulting in poor mechanical properties of the film.	[47]

**Table 3:** Researches on the application of materials containing anthocyanins in meat preservation.

Food Category	Sources of Anthocyanins	Other Main Materials	Comparator	Freshness Indicator Response Factor	Color Changes	Main Results	Main Limitation	References
Pork	Cranberry anthocyanins (ACN), cranberry extract, concentration 600.20 mg/L, ɛ-polylysine (ɛPL)-ACN solution (2 mg/mL) was added to the inner film solution.	ɛPL, potato starch, k-carrageenan, chitosan, gelatin, ZnO-doped carbon dots	Fresh state vs. spoiled state	pH and ammonia	Red to yellowish-green	Indicate pork freshness	The different meat or seafood spoilage processes and changes in ambient temperature and humidity may alter the color response.	[49]
Pork (30 g)	Blueberry anthocyanins (BA), loading 0.03% *w*/*v* in the pads forming solution.	citric acid (CA), and yellow lake (Y), poly (vinyl alcohol)/glycerol/gelatin (PGG)	PGG vs. PGG-CA/PGG-CA-BA-Y	pH, NH_3_	Red to orange to green	Maintain fixed water and suppress the increase of free water; inhibit the gravy loss rate, retain over 95% of pork exudate in the pad, compare with PGG, PGG-CA/PGG-CA-BA-Y maintains higher hardness (183.06 g vs. 207.15 g) and elasticity (0.43 vs. 0.57) on the 7th day, inhibit the increase of pH value, total volatile base nitrogen (TVB-N), and total viable count (TVC), extend the shelf life by <1 d at 4 °C.	CA, BA, and Y increase the surface roughness of pads.	[50]
Pork (10 ± 1 g)	Saskatoon berry anthocyanins (SBA), saskatoon berry extract, purity 221.3 ± 1.09 mg/g, film-forming solution (100 mL) contained 10 mL of SBA solution (0.10–0.25 wt%).	Carboxymethyl cellulose (CMC), and xylan	Xylan-CMC vs. Xylan-CMC-SBA	/	/	Compare with Xylan-CMC, Xylan-CMC-SBA delays pH increase (6.42 vs. <6.19) and inhibits bacterial growth (6.02 logCFU·g^−1^ vs. approximately 4 logCFU·g^−1^) in pork on day 8, maintain pork shelf life for at least 8 days, extend the shelf life by at least 2 d at 4 °C.	As the SBA content increases, the tensile strength and elongation at break of the film decrease.	[51]
Pork (10 g)	*Aronia melanocarpa* anthocyanins (A), *Aronia melanocarpa* extract, A (35 mg) was added to a base solution consisting of 150 mL of solution and 3% (*v*/*v*) glycerol.	Tea polyphenols, sodium alginate and pectin (S/P)	S/P vs. S/P/A	Ammonia	Red to colorless to purple	Inhibit increases in pH (6.68 vs. 6.55 on the 6th day), TVB-N, and TVC. Reduce water loss. Compare with S/P film, S/P/A maintains higher L* (35.8 ± 1.7 vs. 40.6 ± 0.9) values and higher pork hardness (12.06 ± 2.55 vs. 13.83 ± 0.98 N), and lower springiness reduction ratio (24.2% vs. 22.9%) and lower adhesiveness increase ratio (53.7% vs. 46.3%) after 15 days at 4 °C, extend the shelf life by approximately 1 d.	/	[52]
Chicken (500 g)	Grape skin anthocyanins (GSA), grape skin extract, anthocyanin content 1.48 mg/g grape skin.	Mandelic acid, betalains, shikonin, gelling gel	EF vs. GSAF (GSA indicator film (GSAF))	pH	Purplish red to light yellow	Inhibit the increase in pH, TVB-N (21.69 mg/100 g vs. <20 mg/100 g on the 6th day), and thiobarbituric acid (TBARS) at 4 °C, extend the shelf life by approximately 1 d	The moisture content of GSAF is higher than EF.	[53]
Chicken	*Syzygium cumini* anthocyanins, *Syzygium cumini* extract.	Chitosan, 2- hydroxyethyl methacrylate, methacrylic acid, carbon quantum dots	Fresh state vs. spoiled state	pH	Purplish-red to brownish-green	Indicate the freshness of chicken at room temperature.	/	[54]
Yak (50 g)	Black rice anthocyanins (ANS), 0.5 mg/mL in the solution.	Egg yolk immunoglobulin (IGY), fucoidan (F)	ANS vs. IGY40-ANS-F	/	/	Compare with IGY40-ANS-F, ANS inhibits pH increase of yak meat on the 9th day at 4 °C.	/	[55]
Chicken (50 g), and beef (50 g)	Grape skin anthocyanins (GPAs), grape skin extract, 3% (*v*/*v*) in the film-forming solution.	Chitosan (CS), κ-carrageenan (KC), rutin nanoparticles (RUT NPs)	Control vs. CS/0.3%KC/0.3%RUT NPs/3%GPA film (T7)	pH and ammonia	Violet to greenish	Compare with control, T7 active film maintain red meat freshness during 11 days of refrigerated storage at 4 °C, with lower TVC (8 vs. 6.8 log CFU/g), psychrotrophic bacteria count (PBC) (9 vs. 8 log CFU/g), peroxide value (5.73 vs. 4.08 meq/kg), pH (7.72 vs. 6.12), and TVB-N (25 vs. 13.9 mg/100 g).	Only 48 h of testing time, tested under specific experimental conditions. The mechanical properties of films have not been measured in real-world environments.	[56]
Mutton (200 g)	Purple potato anthocyanin, purity 100–120 mg/g, containing 2 g of PAM in 80 mL film solution.	Nanosilver filled gellan gum/sodium alginate (GS/Ag)	GS/Ag vs. GS/Ag-PAM 2 (purple potato anthocyanin microcapsules (PAM))	pH and ammonia	Purplish-red to dark blue	Indicate the freshness of mutton at 4 °C.	Excessive PAM loading caused larger voids and cracks and reduced color-response sensitivity, had less transmittance at 660 nm.	[57]

**Table 4:** Researches on the application of materials containing anthocyanins in aquatic products preservation.

Food Category	Sources of Anthocyanins	Other Main Materials	Comparator	Freshness Indicator Response Factor	Color Changes	Main Results	Main Limitation	References
Shrimp (25.78 ± 1.62 g)	*Aronia melanocarpa* anthocyanins (AMA), *Aronia melanocarpa* extract, containing 0.2 mg/mL AMA in film-forming solution.	Ferulic acid (FA), carvacrol (CL), hydroxypropyl-β-cyclodextrin (HP-β-CD), potato starch (PS)	CK vs. IC-FA-AMA/PS I. FA (CL@HP-β-CD (IC))	pH	Pink to blue	Inhibit increases in pH (>7.6 vs. <7.25), TVC (6.02 ± 0.06 vs. approximately 4.5 log CFU/g), and TVB-N (24.64 vs. approximately 12.5 mg/100 g) after 48 h at 4 °C, extend the shelf life by 12 h.	Improvements in mechanical strength, transparency, and release regulation.	[59]
Shrimp	*Vaccinium vitis-idaea* anthocyanins, *Vaccinium vitis-idaea* extract.	Chitosan/corn starch (CTS/Corn), whey protein isolate, chitosan hydrochloride, carboxymethyl chitosan	CTS/Corn vs. CTS/Corn-FA (CTS/Corn-free anthocyanin (CTS/Corn-FA film))	/	/	Compare with CTS/Corn, CTS/Corn-FA inhibit the increase in pH (7.8 vs. approximately 7.5 on day 6), reduce weight loss (10.23% vs. 8.85% on day 10), inhibit TVB-N production (26.6 vs. 22.9 mg/100 g on day 6), reduce lipid oxidation, suppress microbial growth (7.25 vs. 7.05 log CFU/g) in shrimp during storage at 4 °C, extend the shelf life by 2 d.	The addition of anthocyanins causes cracks in the cross-section of the film and makes the surface rougher.	[60]
Shrimp	Blue honeysuckle berry anthocyanins (BA), Blue honeysuckle berry extract, BA (0.5 g) was added to a solution containing 100 mL of deionized water and 30% glycerol.	ZnCDs, chitosan-gelatin (CG), esterified porous starch	Control vs. CG-BA	pH and ammonia	Dark grayish-purple to dark brown	Delay the increase of pH, TVB-N and TVC values in shrimp at 4 °C, extend the shelf life by <0.5 d.	/	[61]
Shrimp (18 ± 1.5 g)	Jambolan fruit anthocyanins, jambolan fruit extract, 1.70 mg/g of fresh fruit weight, containing 212.04 mg of anthocyanins/g of montmorillonite.	Montmorillonite	Fresh states vs. spoiled state	pH and ammonia	Pink to blue	Increase the stability of anthocyanins through biohybrid.	Further evaluation of nanoclay migration and consumer safety, and optimization of scalability for commercial application.	[62]
Shrimp	Blueberry anthocyanins (AN), blueberry extract, purity 25%, 0.01 g in the films.	Chitosan (CS), cellulose nanocrystal (CNCs)	Control or PE vs. CS-AN-CNCs 9% film	pH	Blue to gray-green	Inhibit TVB-N accumulation (>40 vs. 32.40 ± 0.92 mg/100 g) after 30 h at 4 °C, extend the shelf life by approximately 5 h.	/	[63]
Shrimp (9–11 g)	Rosella extract (RE), anthocyanin content 407.22 μg/g.	Carboxymethyl chitosan, chitosan hydrochloride, konjac glucomannan/zein (KZ)	Blank vs. KZ-R-20 (diluted RE solution (R-20))	pH	Purple to dark blue	Inhibit the increase of TVB-N (22.75 vs. 15 ≤ TVB-N ≤ 20 mg/100 g on day 4) during 4 days of storage at 4 °C, extend the shelf life by approximately 2 d.	The addition of anthocyanins reduces water contact angle.	[64]
Shrimp	Black rice anthocyanins (BRA), black rice extract, 10% *w*/*w* based on chitosan (CS) dry weight.	CS, *Phyllanthus emblica* L. polysaccharides (PEP)	CS vs. CS-BRA-PEP 2	pH	Red to brown	Inhibit the production of TVB-N, microbial growth and reproduction, suppress lipid peroxidation (>1.6 vs. 0.85 mg malondialdehyde/kg on day 5), prevent reductions in myofibrillar protein content and Ca^2+^-ATPase activity during 5 days of storage at 4 °C, extend the shelf life approximately 1 d.	The current testing is mainly con-ducted in the la-boratory. The an-tibacterial perfor-mance test only involves a portion of microorganisms. Long term stability optimization of artificial intelli-gence recognition systems is required.	[41]
Shrimp (25 g)	Red cabbage anthocyanins (RCA), red cabbage extract, containing 184 mg anthocyanins/100 g in extract solution.	Carrageenan/gelatin (Car/Gel), licorice root-derived carbon dots (LCDs), silica nanoparticles extracted from rice straw (SiNPs)	Car/Gel/LCDs@SiNPs vs.Car/Gel/LCDs@SiNPs/RCA	pH	Pink to green	Inhibit pH rise (7.6 vs. 7.4), redness, and TVC increase of shrimp during 21 days of storage at 4 °C.	The incorporation of red cabbage anthocyanins reduced the tensile strength and elastic modulus of the nanocomposite film and increased its water vapor permeability and surface roughness.	[65]
Fish (25 g)	Blueberry anthocyanins (BA), concentration 0.09% (*w*/*v*) in film-forming solution.	Sodium carboxymethyl cellulose (SCMC), xanthan gum, gallic acid	Control vs. SCMC-BA	pH, ammonia	Purplish red to colorless or light yellow	Inhibit the increase of TVB-N (28.70 vs. 23.57 mg/100 g on the 6th day) and suppress TVC increase in sturgeon during 10 days of storage at 4 °C, extend the shelf life by <1 d.	The research focused solely on evaluating the indicating effect of intelligent film on sturgeon freshness.	[66]
Fish (15.0 ± 0.5 g)	Black rice bran anthocyanins (BACNs), black rice bran extract, containing 0 mg and 100 mg of BACNs in 100 mL film forming solution, respectively.	Oxidized chitin nanocrystals (O-ChNCs), gelatin	BACNs-Ch0 vs. BACNs-Ch100	pH	Purple to brown	Reduce the TVB-N (>160 vs. 57.77 mg/100 g) and pH during storage at 25 °C and 75% RH.	The addition of BACNs decreased the tensile strength, high anthocyanin concentrations resulted in excessively dark films.	[67]
Fish (16 g)	Blueberry anthocyanins (ACNs), cyanidin cation 25 wt%, 0.2 mg/mL in film forming solution.	Ovalbumin-sodium alginate complex, plasma-modified corn starch, polyvinyl alcohol (PVA)	NP vs. NP-ACNs (NCS/PVA(NP), untreated corn starch (NCS))	pH	Purplish-red to yellow-green	Inhibit the increase of TVB-N, pH, TBARS, and TVC during 7 days of storage at 4 °C, extend the shelf life about 1.5 d.	The limitations include the need to save energy consumption, simplify preparation methods, optimize experimental parameters.	[68]
Fish	Lingonberry anthocyanins (VVE), lingonberry extract.	*Gelidium amansii* polysaccharides (PC)	PC vs. PC-VVE2	pH and ammonia	Pink to brownish purple	Inhibit the increase in pH, TVB-N, and TVC during 16 days of storage at 4 °C, maintain Grade A quality for up to 12 days, extend the shelf life by 6 d.	This study is limited by small sample size, technical limitations, and insufficient characterization of structural changes in polysaccharides and anthocyanins.	[69]
Fish (50 g)	*Solanum nigrum* L. anthocyanins (SNA), *Solanum nigrum* L. extract, purity 0.73 ± 0.01 mg cyanidin-3-glucoside equivalents/g, adding 0.2% (*w*:*w*) of SNA to film forming solution.	polyvinyl alcohol/fish gelatin (PVA/FG)	PVA/FG vs. PVA/FG-0.2%SNA	pH and NH_3_	Dark pink to colorless	Inhibit the increase in TVC, pH, TBARS value, and TVB-N during 16 days of storage at 4 °C, extend the shelf life by 2 d.	Only a single fish species was studied, and the potential impact of environmental factors on the results and the influence of anthocyanin composition on film properties were not investigated.	[70]
Crabs (125 ± 15 g)	Mulberry anthocyanins (MAE), mulberry extract, the concentration of MAE was 8% in the sensor layer.	Gellan gum, chitosan, polyvinyl alcohol, sodium alginate, pullulan	Fresh state vs. spoiled state	pH	Pink to dark green	Indicate the freshness of the crabs during 12 days of storage at 4 °C.	/	[71]

**Table 5:** Researches on the application of materials containing anthocyanins in processed food preservation.

Food Category	Sources of Anthocyanins	Other Main Materials	Comparator	Freshness Indicator Response Factor	Color Changes	Main Results	Main Limitation	References
Tofu	Camphor fruit anthocyanins, camphor fruit extract, treatment concentration 14 mg/mL.	/	CK negative vs. B_1_	/	/	Inhibit microbial growth in tofu, extend the shelf life by 1 d.	/	[72]
Dried tofu (20 g)	Purple cabbage anthocyanins (PCA), a 10 mL solution containing 80% PCA (0.4% (*m*/*v*)) and 20% CU (0.2% (*m*/*v*)) by volume was added into the composite film substrate.	Curcumin (CU), pullulan, polyvinyl alcohol (PCA), polyethylene (PE), and soybean shell nanocellulose	PE vs. PCA/CU-3	pH	Deep red to deep purple	Suppress the color browning, and the increase of pH, TNB-N and TVC contents of dried tofu during 12 days of storage at 4 °C, extend the shelf life by 4 d.	The prepared film shows insufficient light stability, and no large-scale industrial preparation process has been established.	[73]
Tomato sauce	Red cabbage anthocyanins, anthocyanins extract (1.5 mL, concentration 790.69 ± 2.84 mg/L) was added to label-forming solution.	Cellulose acetate, thymol	Without label vs. with label	pH, NH_3_	Purple to green and then to yellow	Limit pH decline and color changes in tomato sauce; reduce TVC (220 vs. 56 CFU after 30 days at 37 °C, and 207 vs. 27 CFU at 55 °C).	The colorimetric labels stored at 37 °C showed a low stability over time.	[74]
Cheese	Elderberry anthocyanins (EA), EA (0.5 g) was added to 200 mL film-forming solution.	konjac glucomannan (KGM), β-cyclodextrin, oregano essential oil, and chitosan	KGM vs. KEA (EA were incorporated into KGM film (KEA))	/	/	Inhibit the darkening of cheese color, suppress the increase in pH and hardness, reduce weight loss (19.14% vs. 18.14% after 2 days) during 12 days of storage at 25 °C.	/	[75]
Chinese pork meatballs (150 g)	*Rhododendron pulchrum Sweet* anthocyanins (RA), RA (2 g/hg) was added to base solution.	Carrageenan, polyvinyl alcohol (PVA), ε-polylysine (ε-PL)	CP vs. CP-RA-6% ε-PL (carrageenan–PVA(CP))	/	/	Reduce weight loss, inhibit increases in pH, TVB-N, and TVC and suppress lipid oxidation, maintain color, taste, and sensory quality during 14 days of storage at 4 ± 1 °C, extend shelf life by 2 days.	The migration and safety of the composite film have not been evaluated, and the actual application has not been clarified.	[76]

## Data Availability

No new data were created or analyzed in this study. Data sharing is not applicable to this article.

## Abbreviations

The following main abbreviations are used in this manuscript:

MDA	Malondialdehyde
SOD	Superoxide dismutase
ABTS	2,2′-Azino-bis (3-ethylbenzothiazoline-6-sulfonic acid)
CAT	Catalase
TVB-N	Total volatile base nitrogen
TVC	Total viable count
TBARS	Thiobarbituric acid
ROS	Reactive oxygen species
